# Two Cases of Membranous, or Croupous, Laryngitis in Infants, Treated by Intra-Laryngeal Applications, with Recovery

**Published:** 1895-06-15

**Authors:** George Mackern

**Affiliations:** late Visiting Physician to the British Hospital, Buenos Ayres


### Medical Progress and Hospital Clinics.

[*The Editor will be glad to receive offers of co-operation and contributions from members of the profession. All letters should be addressed to* THE EDITOR, THE LODGE, PORCHESTER SQUARE, LONDON, W.]

### TWO CASES OF MEMBRANOUS, OR CROUPOUS, LARYNGITIS IN INFANTS, TREATED BY INTRA-LARYNGEAL APPLICATIONS, WITH RECOVERY.

By GEORGE MACKERN, M.D. Lond., late Visiting Physician to the British Hospital, Buenos Ayres.

The occurrence of a form of pharyngitis and laryngitis, characterised by the existence of membranous exudations (pseudo-membranes), which differ both clinically and pathologically from what is known as diphtheria membrane, can no longer be denied. I have for many years held the view, based on clinical observations, that the ordinary "croup" of children need not necessarily be "septic," in the sense, I mean, of a diphtheria poison, which, though primarily local, sooner or later affects the system through the blood and lymphatics. "In diphtheria," Schech remarks, "a symptom which is always present is swelling of the lymphatic glands; it occurs even in the mildest cases, while in severe ones it attains a most extraordinary intensity."

In membranous or croupous pharyngitis or laryngitis this is by no means the rule, and in the two cases I am about to describe, and in other cases where the usual tracheotomy had been performed, there was no evident infiltration of the glands. This is an important point in the diagnosis, especially where, as in the case of very young children, it is not always possible to remove bits of membrane and make the necessary observations as to whether it is "thick, yellowish, closely adherent to the parts beneath, and not separable from them without bleeding, &c." (Bosworth.) In neither of the following cases was it possible to remove any membrane whatever from the pharynx, and the symptoms of general toxæmia were so slight that in neither case did I consider the system to be seriously affected, except by the usual signs of carbonic acid toxæmia due to the stenosed larynx.

Both these children had been ill for some days, with "croupy" symptoms from the beginning, and I think all observers will agree that diphtheria cannot exist in the larynx of a child, even for a few hours, without there being present, at the same time, serious blood poisoning. More especially is this true if the diphtheria has commenced in the pharynx and spread downwards. In an ordinary case of pharyngeal diphtheria, extension to the larynx, in my experience, means practically septic absorption.

I admit that in both these cases the diagnosis was rendered more certain by the use of the laryngoscope; but I affirm that, even without the use of the mirror, a diagnosis of "not diphtheria" could have been made.

Case I.—A. B., female, aged 13 months, lived in a low, damp district close to the port, where diphtheria is almost endemic. I was called to see her late at night, and found the child almost *in extremis*, and that the medical attendant (an Italian doctor) had left her, saying he could do no more for her. She was pale, and somewhat collapsed; pulse feeble, 110; temperature, 100·5; skin fairly moist, no cold, clammy sweats; respirations frequent and shallow; dyspnœa with marked retraction in epigastric region; almost complete aphonia, with frequent, short, rough, grating cough; no paroxysms of coughing, nor violent inspiratory efforts. On examination, I saw some white pseudo-membranous spots, not larger than apple-pips, on the tonsils and posterior pillars of the fauces, the velum and the posterior pharyngeal wall being free; the mucous membrane was not acutely inflamed in any part, not even round the edges of the patches. The larynx was very small, pale pink in colour, and its interior filled with a whitish deposit (a more exact description was not possible), the epiglottis erect, and free from any deposit whatever.

As regards treatment, the indication was evidently tracheotomy or intubation; but taking into consideration the absence of another medical man, the lateness of the hour, and my distance from home, I determined to clean out the larnyx, and mechanically remove the obstructing masses of deposit or membrane. This I proceeded to do with cotton wool securely wrapped round the end of a metallic laryngeal probe bent to the required angle, and saturated with a 10 per cent. solution of carbolic acid in glycerine. The introduction of the probe well below the level of the cords produced a nasty spasm, and some violent inspiratory efforts, but I was gratified to find quite a number of thick white flakes of membrane sticking to the cotton wool when I removed the probe, and no blood came away with them. The child struggled a little and then uttered a distinct cry, and the respirations at once began to improve.

Before leaving I also touched the pharyngeal spots firmly with the carbolic solution, and ordered hot fomentations to the throat, steaming of the room with a mixture of eucalyptol, tincture of benzoin, and ol. terebinth, and some potass. brom., with a few drops of vini ipecac. to be taken every two or three hours. Nourishment was to be only hot milk with a few drops of brandy every two hours during the night. Next morning I found the child much better, respiration fairly normal, voice still hoarse, temperature 99 degrees, and general appearance of liveliness, so much so that I found it impossible to catch even a glimpse of the larynx with the mirror. The pharyngeal spots disappeared after a few days, and the child remained quite well.

Case II.—E. D., female, aged 18 months, lived in a high, healthy district. Called to see her late at night, and found, as in the former case, that she had been abandoned as "hopeless" by the doctor. As I came into the house it was evident the case was urgent, as the stridor could be plainly heard outside the room. The child was slightly flushed, lying quietly in its mother's arms; temperature 101·2; pulse feeble and about 120; respirations very frequent and noisy; almost complete aphonia, very rough dry cough at frequent intervals. On examination of the pharynx I could only make out one small white spot, not larger than a millet seed, on the posterior right faucial pillar, and with the laryngscope occlusion of the rima glottidis by a white flaky mass. The colour of the larynx generally was a brighter pink than in the former case, and the epiglottis was covered in its inferior and laryngeal surface with a membranous deposit. I applied the same treatment as in the former case, the entrance of the probe into the larynx producing a very violent spasm. On withdrawal, only a small piece of white flaky material remained on the cotton wool, but I must have dislodged a certain amount, as before I left the breathing had improved somewhat, and the attacks of cough were louder and more frequent, which I considered a favourable sign. Steaming and general treatment as in the other case. The immediate improvement in this case was not so pronounced, and I left the house with some misgivings; but next morning I found considerable improvement in the respirations, and the temperature lower. I made another intra-laryngeal application, and repeated it in the afternoon. By the second day, however, the dyspnœa had practically disappeared, and I suspended active local treatment, but continued the general, and the child recovered completely in a few days.

**Remarks.**—In the first case the child had been ill a couple of days, and, although the larynx was much obstructed, yet the case altogether did not seem so "asthenic" as the second, where the child had been ill three or four days, had a higher temperature, and had been subjected to active treatment. In the second case there was no albumen in the urine, in the first case no urine was obtainable. In neither case were the membranes examined microscopically, nor was it the fashion in those days (1891) to make systematic bacteriological examinations.

As regards the intra-laryngeal applications, no doubt the mechanical clearing out of the larynx was largely beneficial, but as I used a 10 per cent. solution of carbolic acid in glycerine on the cotton wool, the germicidal action of the acid must be also taken into consideration. In a third case, which I had seen long previously to this in consultation with Drs. Alston and Pirovano, I got a good view of the larynx, while the child (a boy aged seven years) lay in his cot, with the help of Semior's electric laryngoscope. It was a much more acute case with high temperature, urgent dyspnœa, and great restlessness. The pharynx was quite normal, but the larynx acutely inflamed with white exudations lying chiefly on the ventricular bands and reducing the lumen of the glottic opening to a mere chink. Tracheotomy was immediately performed with great relief to symptoms and complete recovery was rapid, but I think that now I should first try endo-laryngeal applications before resorting to operation.

